# Clinical Significance of the Relationship between Progression-Free Survival or Postprogression Survival and Overall Survival in Patients with Extensive Disease-Small-Cell Lung Cancer Treated with Carboplatin plus Etoposide

**DOI:** 10.1155/2016/5405810

**Published:** 2016-06-29

**Authors:** Hisao Imai, Keita Mori, Nodoka Watase, Sakae Fujimoto, Kyoichi Kaira, Masanobu Yamada, Koichi Minato

**Affiliations:** ^1^Division of Respiratory Medicine, Gunma Prefectural Cancer Center, 617-1 Takahayashinishi, Ohta, Gunma 373-8550, Japan; ^2^Department of Medicine and Molecular Science, Gunma University Graduate School of Medicine, 3-39-15, Showa-machi, Maebashi, Gunma 371-8511, Japan; ^3^Clinical Research Support Center, Shizuoka Cancer Center, 1007 Shimonagakubo, Nagaizumi-chou, Suntou-gun, Shizuoka 411-8777, Japan; ^4^Division of Pharmacy, Gunma Prefectural Cancer Center, 617-1 Takahayashinishi, Ohta, Gunma 373-8550, Japan; ^5^Department of Oncology Clinical Development, Gunma University Graduate School of Medicine, 3-39-15 Showa-machi, Maebashi, Gunma 371-8511, Japan

## Abstract

*Background*. The effects of first-line chemotherapy on overall survival (OS) might be confounded by subsequent therapies in patients with small-cell lung cancer (SCLC). Therefore, by using individual-level data, we aimed to determine the relationships between progression-free survival (PFS) or postprogression survival (PPS) and OS after first-line chemotherapies in patients with extensive disease-SCLC (ED-SCLC) treated with carboplatin plus etoposide.* Methods*. Between July 1998 and December 2014, we analyzed 63 cases of patients with ED-SCLC who were treated with carboplatin and etoposide as first-line chemotherapy. The relationships of PFS and PPS with OS were analyzed at the individual level.* Results*. Spearman rank correlation analysis and linear regression analysis showed that PPS was strongly correlated with OS (*r* = 0.90, *p* < 0.05, and *R* ^2^ = 0.71) and PFS was moderately correlated with OS (*r* = 0.72, *p* < 0.05, and *R* ^2^ = 0.62). Type of relapse (refractory/sensitive) and the number of regimens administered after disease progression after the first-line chemotherapy were both significantly associated with PPS (*p* < 0.05).* Conclusions*. PPS has a stronger relationship with OS than does PFS in ED-SCLC patients who have received first-line chemotherapy. These results suggest that treatments administered after first-line chemotherapy affect the OS of ED-SCLC patients treated with carboplatin plus etoposide.

## 1. Introduction

Lung cancer is the leading cause of cancer-related deaths worldwide [1], and small-cell lung cancer (SCLC) accounts for almost 13% of all new cases [2]. More than half of these patients are diagnosed with extensive disease- (ED-) SCLC [3]. In ED-SCLC cases, chemotherapy alone can palliate symptoms and prolong survival in most patients; in chemoresponsive patients, prophylactic cranial irradiation (PCI) can also palliate symptoms and prolong survival. However, long-term survival is rare in ED-SCLC cases [4, 5]. Although many patients initially achieve clinical remission or disease control with first-line chemotherapy, most subsequently experience disease progression and eventually die of ED-SCLC. The first-line treatment of choice in nonelderly ED-SCLC patients with a good performance status (PS) without cardiorenal dysfunction and poor-risk is 4 cycles of cisplatin plus etoposide or cisplatin plus irinotecan [6–9]. Here, we examined first-line carboplatin and etoposide combination chemotherapy because it is considered as one of the standard first-line chemotherapy regimens in ED-SCLC patients with cardiorenal dysfunction and poor-risk [10]. SCLC refers to a rapidly proliferating tumor that is highly sensitive to chemotherapy. However, rapid emergence of clinical drug resistance has resulted in poor prognosis, with almost all such patients dying within 2 years of the initial diagnosis [3]. For ED-SCLC patients, OS is shorter and options for subsequent chemotherapy are limited.

Progression-free survival (PFS) and OS are two common endpoints in cancer trials. OS is usually preferred, because it is reliable, precise, and meaningful, and it can be easily documented by noting the date of death. However, the effect of first-line treatments on OS might be confounded by subsequent lines of therapy [11]. In contrast, as PFS measurement is quicker and more convenient, it may be easier to assess than OS [12]. If there is a strong correlation between PFS and OS, PFS may be a surrogate endpoint for OS. In non-small-cell lung cancer (NSCLC), an increase in the PFS does not necessarily result in an increase in OS [13], but postprogression survival (PPS) is strongly associated with OS after first-line treatment [14, 15]. Although PFS following first-line chemotherapy has not been validated as a surrogate endpoint for OS, PPS has been shown to be strongly associated with OS after first-line chemotherapy for advanced NSCLC [16–18]. Furthermore, OS can be approximated as the sum of PPS and PFS [11]. A previous report has also demonstrated a strong correlation between PPS and OS after first-line chemotherapy using cisplatin plus irinotecan in nonelderly ED-SCLC patients with a good PS using individual-level data [19]. However, in ED-SCLC patients with a PS of 0–2 with cardiorenal dysfunction who cannot receive cisplatin combination chemotherapy or patients with a PS of 3 treated with carboplatin plus etoposide, the relationship between PPS and OS is unknown. The significance of PPS in ED-SCLC patients treated with carboplatin plus etoposide also remains unclear. Therefore, by using individual-level data, we aimed to determine the relationships between PFS or PPS and OS after first-line chemotherapy for ED-SCLC patients treated with carboplatin plus etoposide.

The present study analyzed the relationships of PFS and PPS with OS in patients with ED-SCLC. The patients recruited for this study had only limited options for subsequent-line chemotherapy. We also explored the prognostic value of baseline and tumor characteristics for PPS.

## 2. Materials and Methods

### 2.1. Patients

Between July 1998 and December 2014, 64 patients with extensive SCLC were treated with carboplatin and etoposide as first-line chemotherapy and were retrospectively enrolled in this study. In our institution, patients aged less than 75 years with good PS and good cardiorenal function are usually given cisplatin plus irinotecan or cisplatin plus etoposide, but patients with a PS of 0–2 with cardiorenal dysfunction who cannot receive cisplatin combination chemotherapy or patients with a PS of 3 are given carboplatin plus etoposide. The inclusion criteria were as follows: histologically or cytologically confirmed SCLC; ≤74 years of age at the time of chemotherapy; Eastern Cooperative Oncology Group PS of 0–3 at the beginning of the first-line treatment; and disease progression after first-line treatment. Tumor response was not evaluated in one case. One patient was excluded from the analyses to maintain uniformity in patient background characteristics. Thus, data from 63 patients were analyzed. For this type of study, formal consent was not required.

### 2.2. Treatments

The study patients were treated with carboplatin [area under the curve (AUC) = 5 for 1 day, followed by a pause of 21 days] and etoposide (80–100 mg·m^−2^·day^−1^ on days 1, 2, and 3, followed by a pause of 21 days). This cycle was repeated every 3-4 weeks for a maximum of 4 courses. After chemotherapy, PCI (25 Gy/10 fractions) was administered to patients with a complete or near-complete response, as shown by a scar-like shadow on a chest computed tomography (CT), if the treating physician recommended it.

### 2.3. Assessment of Treatment Efficacy

The best overall response was recorded as tumor responses. Radiographic tumor responses were evaluated according to the Response Evaluation Criteria In Solid Tumors, ver. 1.1 [20]: complete response (CR), disappearance of all target lesions; partial response (PR), ≤30% decrease in the sum of the target lesion diameters with the summed baseline diameters as a reference; progressive disease (PD), ≤20% increase in the sum of the target lesion diameters with the smallest sum observed during the study serving as reference; and stable disease (SD), insufficient shrinkage to qualify as PR and insufficient expansion to qualify as PD. PFS was calculated from the start of treatment to the date of PD or death from any cause. OS was recorded from the first day of treatment until death or was censored on the date of the last follow-up consultation. PPS was recorded as the time from tumor progression until death or was censored on the date of the last follow-up consultation.

### 2.4. Treatment-Free Interval

In this study, we defined treatment-free interval (TFI) as the period from the date of completion of first-line treatment to first relapse. When prophylactic cranial irradiation (PCI) was performed as first-line treatment, the date of completion of first-line treatment was defined as the last day of the treatment. Since TFI is known to be a predictive factor of second-line chemotherapy [21, 22], we analyzed patients according to TFI. In many trials, SCLC cases—with a TFI of ≥90 days—that relapsed were defined as sensitive relapse cases. We used the same definition for sensitive relapse cases in this study.

### 2.5. Statistical Analyses

To examine whether PFS or PPS was correlated with OS, we used Spearman rank correlation analysis and linear regression analysis. To identify possible prognostic factors for PPS, a proportional hazards model with a stepwise regression procedure was applied. Hazard ratios (HR) and 95% confidence intervals (CI) were estimated using this model. Because the HR is defined for a 1-unit difference, some factors were converted to an appropriately scaled unit. PPS values were compared using the log-rank test. A *p* value of ≤0.05 was considered significant for all tests. The two-tailed significance level was also set at 0.05. All statistical analyses were performed using JMP version 11.0 for Windows (SAS Institute, Cary, NC, USA).

## 3. Results

### 3.1. Patient Characteristics and Treatment Efficacy

Of the 63 patients included in the analyses, 62 patients died; the median follow-up time was 8.2 months (range, 0.3–58.2 months).  Table 1 shows the characteristics of the 63 patients (median age, 67 years; range, 50–74 years) included in the study. Target lesions were evaluated in all cases. One, 41, 7, and 14 patients showed CR, PR, SD, and PD, respectively. The response rate was 66.7% and the disease control rate was 77.8%.

Of the 63 patients who exhibited relapse after the first-line chemotherapy, 20 did not receive further chemotherapy. The other 43 patients received subsequent chemotherapy after completing their first-line chemotherapy. Among the 63 patients, the median number of follow-up therapeutic regimens was 1 (range, 0–5 regimens).  Table 2 shows the chemotherapy regimens administered in cases that showed relapse after the first-line chemotherapy regimen. Amrubicin was the most common second-line chemotherapy agent, and carboplatin plus irinotecan was the most common third-line chemotherapy agent.

**Figure 1:**
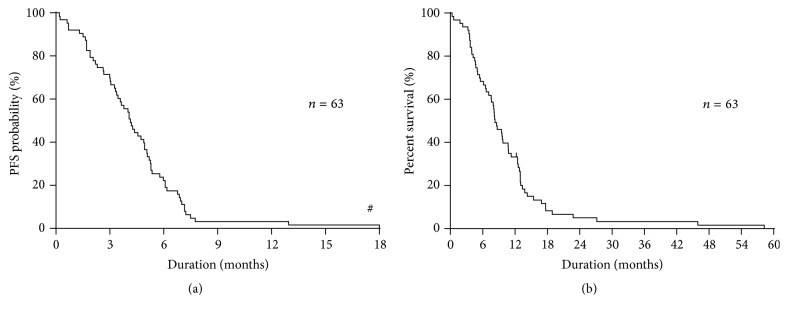
(a) Kaplan-Meier plots showing progression-free survival (PFS). Median progression-free survival: 4.1 months. ^#^Outlier of one case exists. (b) Kaplan-Meier plots showing overall survival (OS). Median overall survival: 8.2 months.

The median PFS and OS were 4.1 months and 8.2 months, respectively (Figures 1(a) and 1(b)).

### 3.2. Relationship between OS and PFS and PPS

**Figure 2:**
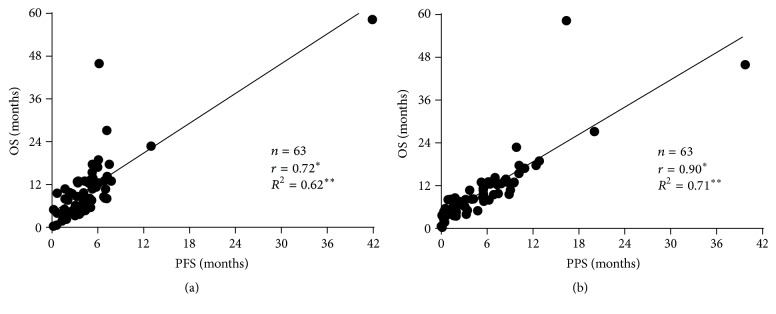
(a) Correlation between overall survival (OS) and progression-free survival (PFS). (b) Correlation between overall survival (OS) and postprogression survival (PPS). ^*∗*^The  *r* values represent Spearman's rank correlation coefficient. ^*∗∗*^The  *R* ^2^ values represent linear regression.

**Figure 3:**
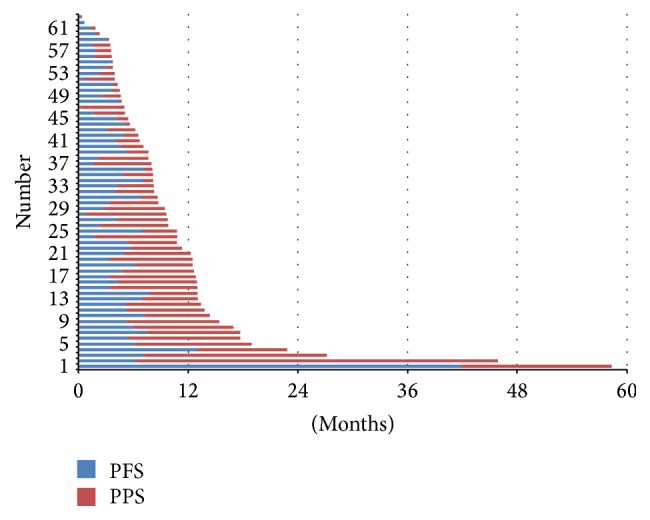
Progression-free survival (PFS) and postprogression survival (PPS) in the overall population.

The relationship between OS and PFS and PPS is shown in Figures 2(a) and 2(b), respectively. Spearman's rank correlation coefficient and linear regression revealed that PPS was strongly associated with OS (*r* = 0.90, *p* < 0.05, and *R* ^2^ = 0.71), whereas PFS was moderately correlated with OS (*r* = 0.72, *p* < 0.05, and *R* ^2^ = 0.62). Furthermore, Figure 3 shows the PFS and PPS of the entire population.

### 3.3. Factors Affecting PPS

**Figure 4:**
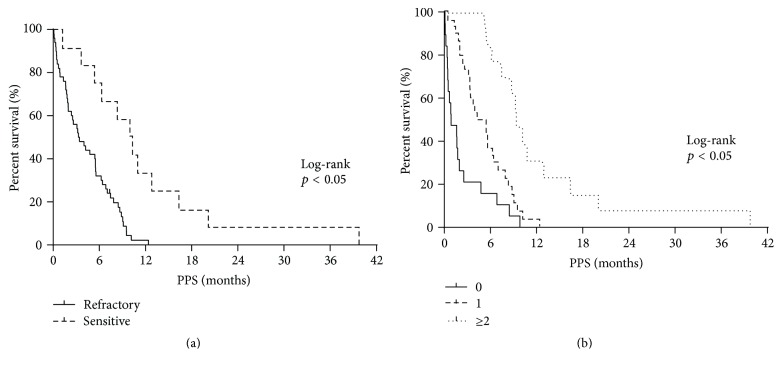
(a) Kaplan-Meier plots showing postprogression survival (PPS), according to the type of relapse. Refractory relapse, median = 3.3 months; sensitive relapse, median = 10.0 months. (b) Kaplan-Meier plots showing postprogression survival (PPS), according to the number of regimens after progression. No further regimen, median = 0.8 months; 1 regimen, median = 4.8 months; ≥2 regimens, median = 9.5 months.

PPS was strongly associated with OS. Therefore, the association between PPS and various clinical factors was assessed. In the univariate analysis (Table 3), the number of courses of first-line treatment administered, PS at the end of first-line treatment, PS at the beginning of second-line treatment, and type of relapse (refractory/sensitive) as well as the best response at the first-line treatment, the best response at the second-line treatment, administration of platinum rechallenge, administration of amrubicin, administration of topotecan, and the number of regimens administered following relapse after the first-line chemotherapy were found to be associated with PPS (*p* < 0.05). Next, a multivariate analysis for PPS (Table 4) revealed that the type of relapse (refractory/sensitive) and number of regimens administered following disease progression after the first-line chemotherapy were significantly associated with PPS (*p* < 0.05). The log-rank tests confirmed that PPS was significantly associated with the type of relapse (refractory/sensitive) as well as the number of regimens employed following disease progression after the first-line chemotherapy (*p* < 0.05; Figures 3(a) and 3(b)). Based on the type of relapse (refractory/sensitive), sensitive relapse cases showed median PPS of 10.0 months, which was longer than that of their counterparts, who had a refractory relapse of 3.3 months (log-rank test, *p* < 0.05; Figure 4(a)). According to the number of regimens administered following disease progression after the first-line chemotherapy, the median PPS for those who were not given additional regimens was 0.8 months; for those with 1 additional regimen, the median PPS was 4.8 months; for those with ≥2 regimens, the median PPS was 9.5 months (log-rank test, *p* < 0.05; Figure 4(b)). These results remained consistent after adjustment using the Cox proportional hazards models (Table 4).

## 4. Discussion

We examined the relationships of OS with PFS and PPS at the individual level in ED-SCLC patients treated with carboplatin plus etoposide. PPS was strongly associated with OS, whereas PFS was moderately correlated with OS. In addition, the type of relapse (refractory/sensitive) after first-line treatment and the number of regimens employed following disease progression after the first-line chemotherapy independently affected PPS. To our knowledge, this is the first report of individual-level factors that affect PPS in ED-SCLC patients after first-line carboplatin plus etoposide.

The validity of surrogate endpoints has been previously determined through meta-analyses [23, 24]. In recent years, biostatisticians have proposed various measures for validating surrogate endpoints [25, 26]. Although PFS is a potential surrogate endpoint for OS in ED-SCLC [27, 28], its validity remains controversial. Broglio et al. recently focused on PPS, which they termed survival postprogression (defined as OS minus PFS), in a hypothetical clinical trial setting under the assumption that treatment affected PFS but not PPS [11]. Recently, a clinical trial reported that PPS was strongly associated with OS after first-line chemotherapy for advanced NSCLC [14, 15], and we have previously reported the significance of PPS for advanced NSCLC and ED-SCLC based on an analysis of individual patients [16–19].

In contrast with the findings of previous studies [27, 28], we did not find PFS to be a surrogate endpoint for OS in our ED-SCLC patients, although PPS was not evaluated in the previous studies. We analyzed our results pertaining to first-line therapy, which suggested that PFS did not adequately reflect OS in such settings. We found that PFS was much shorter than PPS; thus, PPS was closely related to OS—the relationship was linear. The fact that PPS accounted for the majority of OS suggests that the chemotherapy used was not sufficiently effective for PFS to be a significant component of OS. In a disease with a dismal prognosis like ED-SCLC, there is no doubt that OS should remain the primary endpoint for demonstration of efficacy, both in first and in subsequent lines. From this point of view, the relevance of the analysis of correlation of PFS and PPS with OS is not substantial for design of clinical trials, compared to other solid tumors characterized by a longer life expectancy and by the availability of a higher number of effective lines of treatment. Thus, in clinical trials where patients are expected to have a short PFS after first-line chemotherapy, for example, those with ED-SCLC, as was the case in our study, factors that affect PPS need to be considered.

Based on trial-level data for advanced NSCLC, long PPS is associated with a good PS and the use of first-line monotherapy including a molecular targeted agent [14]. Studies based on individual advanced NSCLC patients revealed that long PPS was associated with the PS at the beginning of second-line treatment, the best response at the second-line treatment, and the number of regimens administered following disease progression after the first-line chemotherapy [16]. Furthermore, studies based on individual ED-SCLC patients treated with cisplatin plus irinotecan revealed that long PPS was associated with the best response at the second-line treatment and the number of regimens administered following disease progression after the first-line chemotherapy [19]. To date, however, no predictive factors for PPS in ED-SCLC cases treated with carboplatin plus etoposide have been identified. We studied the prognostic value of baseline factors for PPS in individual ED-SCLC patients. We found that the type of relapse after first-line carboplatin plus etoposide treatment and the number of regimens administered following disease progression after the first-line chemotherapy were strongly associated with PPS in those settings. Moreover, we confirmed the significance of these relationships using log-rank tests. Our findings suggest that cases of sensitive relapse result in prolonged PPS after disease progression following the first-line chemotherapy. These patients are also likely to be able to continue chemotherapy and achieve prolonged PPS, which is associated with a longer OS. A previous study reported that long PPS was associated with the best response at the second-line treatment and the number of regimens administered following disease progression after the first-line chemotherapy [19]. Meanwhile, this study revealed that long PPS was associated with the type of relapse and the number of regimens employed following disease progression after the first-line chemotherapy. Although the number of regimens administered following disease progression after the first-line chemotherapy is consistent with previous study results, the relapse type was not examined in the previous report [19]. The number of treatment regimens employed following disease progression after the first-line chemotherapy probably reflects the increasing number of available drugs, such as amrubicin, irinotecan, and topotecan, which are available as second- or third-line chemotherapy for ED-SCLC. In fact, several different agents were used to treat our patients (Table 2).

This study has several limitations. First, the sample size was relatively small. However, because relatively few ED-SCLC patients are treated with first-line carboplatin and etoposide at our institution, this limitation is difficult to overcome, especially as patients with similar background characteristics are needed. Nevertheless, our institution treats the largest number of such cases relatively, and the practice policy is largely uniform simply because this is a single institution. There is of course some bias, but understanding the nature of this bias ensures that the results are still meaningful. In a future study, we intend to include a larger patient cohort, and more detailed examination is warranted. Second, we could not thoroughly evaluate treatments following disease progression after the second-line chemotherapy, although only a few patients received third-line or subsequent chemotherapy. Third, since different treating physicians documented patient responses, the timing of evaluation of PFS and tumor response rates may have been less accurate than the case if only a single physician had documented all responses. Fourth, there is censored survival data. However, it does not influence our conclusion. Even if the death event of the patients does not occur, there are no changes in PFS. Furthermore, PPS and OS are extended, and PPS was more strongly associated with OS.

In conclusion, PPS has a greater impact on OS than PFS in ED-SCLC patients who have received first-line carboplatin plus etoposide treatment. Additionally, the type of relapse after first-line treatment and the number of additional regimens administered after the first-line treatment are significant independent prognostic factors for PPS. These results suggest that treatments administered after first-line chemotherapy affect the OS of ED-SCLC patients. However, larger multicenter studies are needed to validate these conclusions in other patient populations and clinical settings.

## Figures and Tables

**Table 1:** Baseline patient characteristics.

Characteristic	Number
Sex	
Male/female	52/11
Median age at the time of treatment (years)	67 (50–74)
Performance status	
0/1/2/≥3	6/23/19/15
Smoking history	
Yes/no	63/0
Number of first-line chemotherapy courses	
1/2/3/4/≥5	6/13/4/40/0
Median (range)	4 (1–4)
Number of regimens administered following disease progression after the first-line chemotherapy	
0/1/2/3/≥4	20/30/10/2/1
Median (range)	1 (0–5)
Brain metastases at initial diagnosis	
Yes/no	25/38
Prophylactic cranial irradiation	
Yes/no	1/62
Type of relapse	
Sensitive/refractory	12/51
Median follow-up period [months] (range)	8.2 (0.3–58.2)

**Table 2:** Chemotherapy regimens administered following disease progression after the first-line chemotherapy.

	Second line	More than third line	Total
CBDCA + etoposide rechallenge	1	1	2
CBDCA + irinotecan	15	9	24
Amrubicin	25	5	31
Topotecan	2	3	6
Others	0	0	0

CBDCA: carboplatin.

**Table 3:** Univariate Cox regression analysis of baseline patient characteristics for postprogression survival.

Factors	Postprogression survival		
	Hazard ratio	95% CI	*p* value
Gender			
Male/female	1.60	0.84–3.37	0.15
Age at the beginning of first-line treatment (years)	1.00	0.96–1.04	0.79
PS at the beginning of first-line treatment	1.11	0.86–1.42	0.40
Number of courses of first-line treatment administered	0.72	0.56–0.93	**0.01**
Best response at first-line treatment			
PR/non-PR	0.39	0.22–0.71	**<0.05**
Non-PD/PD	0.55	0.30–1.08	0.08
PS at the end of first-line treatment	2.09	1.56–2.74	**<0.001**
Brain metastases at initial diagnosis			
Yes/no	1.04	0.61–1.73	0.88
Type of relapse			
Refractory/sensitive	4.28	2.03–10.25	**<0.001**
Age at the beginning of second-line treatment (years)	0.99	0.95–1.04	0.90
PS at the beginning of second-line treatment	2.49	1.59–3.92	**<0.001**
Best response at second-line treatment			
PR/non-PR	0.34	0.17–0.67	**<0.05**
Non-PD/PD	0.22	0.09–0.56	**<0.05**
Administration of platinum rechallenge			
Yes/no	0.51	0.28–0.88	**<0.05**
Administration of AMR			
Yes/no	0.39	0.22–0.67	**<0.001**
Administration of TOP			
Yes/no	0.32	0.11–0.77	**<0.05**
Reason for carboplatin + etoposide administration			
Cardiorenal dysfunction/poor PS	0.82	0.49–1.36	0.44
Number of regimens administered following disease progression after the first-line chemotherapy	0.36	0.24–0.51	**<0.001**

95% CI: 95% confidence interval; PS: performance status; PR: partial response; PD: progressive disease; AMR: amrubicin; TOP: topotecan.

Bold *p* values are statistically significant (*p* < 0.05).

**Table 4:** Multivariate Cox regression analysis of PS at the end of first-line treatment, type of relapse, administration of platinum rechallenge, administration of AMR, and number of regimens administered following disease progression after the first-line chemotherapy for postprogression survival.

Factors	Postprogression survival		
	Hazard ratio	95% CI	*p* value
PS at the end of first-line treatment	1.39	0.98–1.95	0.06
Type of relapse			
Refractory/sensitive	2.24	1.00–5.73	**0.04**
Administration of platinum rechallenge			
Yes/no	0.93	0.33–2.75	0.89
Administration of AMR			
Yes/no	1.12	0.41–3.26	0.82
Number of regimens administered following disease progression after the first-line chemotherapy	0.45	0.19–0.91	**0.02**

95% CI: 95% confidence interval; PS: performance status; AMR: amrubicin.

Bold *p* values are statistically significant (*p* < 0.05).
